# Modelling the significance of psychological, social, and situational factors on work efficiency and the preference for working from home in Southeast Asia

**DOI:** 10.1016/j.heliyon.2023.e17561

**Published:** 2023-06-23

**Authors:** Wong May Yee, Abdullah Al Mamun, Zhong Xueyun, Wan Mohd Hirwani Wan Hussain, Qing Yang

**Affiliations:** a UKM - Graduate School of Business, Universiti Kebangsaan Malaysia, 43600, UKM Bangi, Malaysia; b Faculty of Economics & Management, Universiti Kebangsaan Malaysia, 43600, UKM Bangi, Malaysia

**Keywords:** Autonomy, Covid-19, Digital capability, Preference for working from home, Work efficiency

## Abstract

The shift in work paradigm owing to the implementation of new policies in the developing countries of Southeast Asia to reduce the spread of COVID-19 has created new challenges for both employers and employees. The study aimed to address the lack of extensive research on the effects of psychological, social, and situational factors on the work-from-home shift in Southeast Asia. This study incorporates the job characteristic theory, emphasizing how specific job characteristics influence motivation and performance. The study emphasizes the importance of creating an innovative and supportive work environment, improving digital capabilities, and promoting sustainable development through high-skill jobs to enhance remote employees’ productivity. Valid responses from 288 full-time employees who have the option to work remotely were collected through online survey. The findings indicate that self-discipline, digital capability, and perceived organizational support significantly influence the preference for remote work. Managers should focus on motivating employees, providing support, and establishing a digital infrastructure to maximize productivity. Training and recruitment strategies should adapt to the changing work culture, while social support is crucial for encouraging innovative problem-solving. Trusting employees with autonomy and providing appropriate technologies fosters collaboration, efficiency, and creativity in different work settings.

## 1 Introduction

The COVID-19 pandemic has drastically altered the way work is conducted [1]. Before the pandemic, remote working was not a popular practice, and it was mainly available to those with high incomes [2]. Data from Global Workplace Analytics [3] show that 75% remote workers had an income of more than $65,000 per year. This implies that most people had no experience with remote working, making it difficult for them and their employers to transition to this new way of working. The pandemic has forced millions of people to become remote workers, resulting in a global experiment on remote work [4]. Remote work has now become the norm. The countries in Southeast Asia, most of which are developing countries, were not used to this type of work before the pandemic [5]. The manufacturing industry, which is labor-intensive and requires physical attendance at the workplace, is the dominant industry in developing countries, such as China and Malaysia [6,7]. According to the study conducted in Southeast Asia, work efficiency and the preference for working from home are important for workers and organizations [8]. The COVID-19 pandemic has caused a major shift in where we work, when we work, and how we work. Employers that promote hybrid work arrangements and provide the flexibility for employees to work anywhere and anytime are ahead of the curve. These employers are likely to have better employee attraction, retention, and satisfaction in the long run, which could positively impact the business. Hybrid work arrangements are expected to improve productivity and creativity. As employers adapted to offering hybrid work arrangements, they had to change their work practices to better manage productivity during the pandemic, which includes establishing meeting/email-free times for the team or company, using productivity tools, setting aside time on the calendar for individual focused work, establishing clear working hours for work-life balance, and reducing meeting times to 25–45 min to allow for breaks in between meetings [8].

The outbreak of the pandemic and the need to work from home have created several challenges [1] owing to psychological, social, and situational factors. These include self-discipline [9], innovativeness [10], social interaction [11], perceived organizational support [12], digital capability [13], and autonomy [14]. These factors impact both work efficiency and employees’ preference for working from home. Nevertheless, there is a lack of empirical research regarding the Southeast Asians’ preferences to work from home and their work efficiencies during the COVID-19 pandemic [15].

The 5S methodology (*sort, set in order, shine, standardize, and sustain*), a tool commonly used for continuous improvement in manufacturing, underlines self-discipline (“Shitsuke”) as the final step in the successful implementation of a process [16]. In an organizational context, self-discipline is crucial for defining success in terms of continuous improvement. In the personal context, self-discipline is important in defining one’s work efficiency. Empirical research by Wang et al. [9] showed that employees with less self-discipline face more challenges when they work from home. They are unable to overcome work-home interference, causing work-life conflict and a reduction in job performance [17]. Apart from self-discipline, innovative behavior is a factor that affects work efficiency. For instance, innovativeness allows employees to come up with unique solutions combined with inputs for a better result [18]. When employees face problems while working from home, their creativity and problem-solving skills enable them to maintain high efficiency at work. Maslow’s hierarchy of needs explains that providing basic human needs is essential for the employees’ well-being; therefore, having social interactions [11] and feeling supported by their organization [12] are essential for a successful work environment. Positive communication between colleagues, whether work-related or not, has a beneficial effect on the job [11]. This, in turn, encourages employees to be more productive as they become motivated.

Additionally, perceived organizational support increases employees’ motivation, creating employees’ needs to fulfil companies’ objectives [19]. When the Chinese and Malaysian governments announced a lockdown or movement control order (MCO), the employees who perceived organizational support continued with their jobs remotely. According to the empirical study by Chaacha and Botha [20], employees who do not receive support from their managers influence other employees to leave the organization. In addition, employees who have better digital capabilities prefer to work from home because they can solve the problems they face. Employees’ productivity is used to measure their key performance. They are unable to perform their duties and responsibilities if they lack the skills and expertise to manage digital technologies [13]. Finally, the employees who can perform their tasks autonomously are more efficient at work. They can organize their work and ensure that the work is completed within the allocated time. Employees’ performance is defined by their degree of autonomy and their social interactions that ease work constraints [21].

This study concentrates on Malaysian and Chinese full-time employees’ preference to work from home for addressing the gaps in the existing literature on factors affecting work efficiency in the developing Southeast Asian countries. Jacks [15] suggested further empirical research to assess personal and organizational productivity when working remotely in addition to the potential acceptance of working from home. This study examined the psychological, social, and situational factors to measure their impact on work efficiency, and to evaluate the correlation between work efficiency and preference for working from home. The theoretical basis of this study, the hypotheses development, and the methodology used, are presented in the following sections. Subsequently, the results are discussed in relation to the extant literature. Finally, the implications and limitations of this study are discussed.

## 2 Literature review

### 2.1 Theoretical foundation

Maslow [22] proposed that motivation is determined by a hierarchy of needs, beginning with fundamental physiological needs (food, water, and air), followed by safety needs (physical and psychological safety), belonging needs (a place in a group and social support), esteem needs (recognition, achievements, and status), and self-actualization needs (the ability to live up to one’s full potential). Conforming to Maslow's [22] hierarchy of needs, organizations can help fulfill employees’ needs by providing autonomy, and demonstrating compassion and support for individual differences. When examining the literature on work efficiency through the lens of Maslow's [22] hierarchy of needs, workplace factors influencing work efficiency, such as self-discipline, innovativeness, social interaction, autonomy, and perceived organizational support, fit into one of the five categories of needs, which corresponds to a factor that promotes motivation. Employees with self-discipline and self-efficacy are confident because they are capable. They have mastered their jobs and achieved their full potential [23]. Combining Maslow's [22] hierarchy of needs into a single guiding framework allows for the creation of a system that prioritizes the synthesis of literature surrounding workplace factors that impact work efficiency and the preference for remote working.

Job characteristic theory, proposed by Hackman et al. [24], focuses on the relationship between job characteristics and employee motivation, satisfaction, and performance. According to job characteristic theory, specific job characteristics can influence an individual’s psychological states, which in turn impact their motivation and overall job outcomes. The job characteristic theory identifies five core job characteristics: skill variety (the extent to which a job requires different skills and tasks), task identity (the degree to which a job involves completing a whole and identifiable piece of work), task significance (the impact and importance of a job on others or the organization), autonomy (the level of independence and decision-making authority in performing the job), and feedback (the extent to which an employee receives clear information about their performance). These characteristics are believed to contribute to experienced meaningfulness, responsibility, and knowledge of results, which can enhance motivation and job satisfaction. Job characteristic theory provides a theoretical basis for understanding how autonomy and other job-related factors influence employee motivation and performance. The grouping of influencing factors into three categories, namely psychological, social, and situational, can be explained based on their distinct characteristics and theoretical foundations. Psychological factors encompass internal aspects of an individual’s motivation and cognition, such as self-discipline and self-efficacy, which align with Maslow’s self-actualization needs. Social factors pertain to interpersonal relationships and social dynamics within the workplace, with social interaction and belongingness needs corresponding to the desire for connection and support. Situational factors, on the other hand, involve external circumstances and organizational conditions, including autonomy and perceived organizational support, which impact work efficiency. This grouping aligns with job characteristic theory [24], which emphasizes how specific job characteristics influence motivation and performance. By categorizing influencing factors into these three groups, we gain a comprehensive framework that incorporates multiple theories to understand the complex interplay between individual needs, social dynamics, and organizational factors in driving work efficiency.

Wong et al. [25] noted that despite the implementation of policies to promote social distancing and remote work during the pandemic, certain sectors, such as agriculture, hospitality, and retail, still have limited options for working from home. In contrast, other industries, such as administration, technology, management, finance, and engineering, are more conducive to remote work. The efficiency of and preference for working from home among employees in such sectors can be influenced by psychological, social, and situational factors. According to Maslow’s theory, motivation is determined by an individual’s hierarchy of needs, implying that even two people with the same job may have different needs [26]. Employees who work from home may have different experiences with work efficiency and preferences for continuing to work from home. This study therefore developed a framework for measuring employees’ work efficiency and preferences for working from home.

### 2.2 Work efficiency and preference for working from home

Work efficiency refers to the level of productivity and effectiveness with which the tasks are accomplished. It encompasses factors such as time management, task prioritization, and the ability to produce high-quality outputs in a timely manner [23]. When considering the preference for working from home, it refers to the inclination or desire of individuals to perform their work remotely, outside of a traditional office setting. The preference for working from home has gained significant attention in recent years, driven by various factors. One reason is the increasing availability of remote work technologies and flexible work arrangements. Working from home offers benefits such as flexibility in work hours, reduced commuting time and costs, and a better work-life balance. It provides individuals with the opportunity to create a customized work environment that suits their needs and preferences, potentially leading to increased job satisfaction and overall well-being.

Work efficiency and the preference for working from home are interconnected. The flexibility and autonomy provided by remote work can contribute to improved work efficiency. Working from home allows individuals to design their work environment and schedule in a way that enhances their productivity and focus. It eliminates potential distractions or interruptions present in a traditional office setting, thereby potentially increasing concentration, and task completion rates. While work productivity and work effectiveness are also important measures, work efficiency specifically focuses on the ability to optimize resources (such as time and effort) to achieve desired outcomes [15]. In the context of working from home, the emphasis is often on how efficiently individuals can manage their time, tasks, and responsibilities in a remote setting. This includes factors such as minimizing time wastage, maintaining focus, and effectively utilizing available resources to achieve desired results and people who believe they can complete their task efficiently from home are more likely to prefer working from home.

### 2.3 Self-discipline

Employees with a high level of self-discipline are dependable, organized, hardworking, and responsible [27]. They also require less guidance and direction from their managers. They have better self-control, can prioritize tasks, and be focused and persistent without diverting their attention, regardless of their surrounding environment [28]. Self-discipline is a personality trait that is continuously exhibited by the employees [29]. Since highly self-motivated employees are not affected by their surroundings, and they can remain focused and accomplish their tasks regardless of their location, they will likely prefer working from home. Wang et al. [9] conducted an interview in China to study the work from home perspectives and commented that a lack of self-discipline in the employees who work from home will lead to procrastination and cyber-loafing, which causes inefficiency at work. On the other hand, if employees have self-discipline, inefficiencies will not occur, and the employees may be more willing to work from home. Hence, based on the above discussion, this study proposes H1a and H1b as follows.

Hypothesis 1a (H1a). Self-discipline is positively related to work efficiency.

Hypothesis 1b (H1b). Self-discipline is positively related to the preference for working from home.

### 2.4 Innovativeness

Innovative employees introduce, create, and apply new ideas to enhance performance whereby they explore and exploit the available opportunities to generate new ideas; they also allow criticism and receive feedback before putting the idea into practice [10]. According to Van Zyl et al. [30], organizations that exhibit innovative behavior have a higher performance level suggesting that they are more innovative. Therefore, employees’ innovative behavior increases work efficiency by helping in developing creative solutions. Since innovativeness is crucial, especially to achieve a breakthrough in technology and to allow new interdisciplinary combinations [31], creativity can help employees overcome the difficulties they face while working from home. Often, innovative behavior involves group discussions and brainstorming sessions before the results are obtained; this means that although innovativeness allows employees to increase their work efficiency by combining creative solutions [18], they still need to be engaged, prepared, and creative. Hence, employees prefer to work from home if they are creative enough to find the solutions to the challenges through group discussions and brainstorming. Based on the above discussion, this study proposes H2a and H2b as follows.

Hypothesis 2a (H2a). Innovativeness is positively related to work efficiency.

Hypothesis 2b (H2b). Innovativeness is positively related to the preference for working from home.

### 2.5 Social interaction

Employees’ social interaction includes their participation, engagement, and affection in the organization; it can be either work- or non-work-related [11]. Social interactions promote a sense of belonging. Advances in communication technology has made communication and information sharing cheap, reliable, and easy [21]. This can increase employees’ work efficiency by aiding their contacts with colleagues and helping in retrieving information easily. In addition, they will not be isolated as they can easily reach out to others via computers or mobile phones. However, advances in communication technology do not always benefit employees. If employees are working from home, but there is insufficient instruction, feedback, and social interaction, it could cause employee exhaustion with work from home practice [25]. Thus, employees who remain socially interacting with their colleagues in work or non-work-related matters will not only increase their work efficiency but also increase their preference to work from home because they can easily reach out to their colleagues, regardless of their work location. Therefore, based on the above discussion, this study proposes H3a and H3b as follows.

Hypothesis 3a (H3a). Social interaction is positively related to work efficiency.

Hypothesis 3b (H3b). Social interaction is positively related to the preference for working from home.

### 2.6 Digital capability

The productivity of employees who work remotely is influenced by their digital skills, including their aptitude to use digital technology for the development of new products, services, or procedures, and their ability to effectively utilize digital technology for everyday tasks [13]. With the development of technologies, employees’ digital capability has become a job requirement as it enables individuals and organizations to remain competitive. Furthermore, the pandemic has required employees to have high capability in technology to ensure productivity even when they work remotely. To be able to work from home, employees need a home office setup that is difficult in the absence of digital capability [32]. Afrianty et al. [13] confirmed that higher digital capability is positively associated with work efficiency. Digital capability refers to the skills and proficiency of lecturers in using digital technology while working from home during the pandemic. The findings indicated that lecturers with stronger digital capabilities exhibited greater productivity in completing their tasks. This highlights the importance of developing digital skills to enhance work efficiency in remote work settings. Additionally, employees with high digital capability will be able to overcome challenges and be more efficient when working from home; therefore, employees with high digital capability will prefer to work from home. Thus, based on the above discussion, we propose H4a and H4b as follows.

Hypothesis 4a (H4a). Digital capability is positively related to work efficiency.

Hypothesis 4b (H4b). Digital capability is positively related to the preference for working from home.

### 2.7 Autonomy

Autonomy is not only a part of psychological needs and a form of motivation [33], but also a form of psychological freedom [34]. Employees with a high level of autonomy are better at self-management [35] and have greater flexibility [36]; they readily assist the organization when there is a lack of resources [37]. Autonomy is a central component of Job Characteristic Theory, as it is believed to enhance motivation and job satisfaction [24]. Therefore, if the employees are not limited by other factors, they can organize their work and ensure that the task is completed within the set timeframe. In addition, when work culture undergoes a suddenly shift, such as when policies are implemented to promote social distancing and employees are required to work from home [25], those with a high level of autonomy adjust to the change quickly. Their self-management characteristic enables job completion, even in the absence of constant supervision by their managers. Employees’ performance is defined by their degree of autonomy and social interactions to ease limitations at work [21]. When employees are highly autonomous, they do not need close supervision to do their work efficiently.

Hypothesis 5a (H5a). Autonomy is positively related to work efficiency.

Hypothesis 5b (H5b). Autonomy is positively related to the preference for working from home.

### 2.8 Perceived organizational support

Employees perceived organizational support increases their dedication towards their organization; it creates employees’ need to fulfill the company’s objectives [19]. Once their needs are fulfilled, they become more efficient at work. According to Mutonyi et al. [31], organizations’ support for employees regarding autonomy is likely to lead to positive outcomes in terms of employees’ innovative behavior and job performance [12]. Furthermore, Chaacha and Botha [20] suggest that a lack of support from managers could cause other employees to leave the organization. Therefore, providing employees with the option to work from home is likely to be seen as a positive change by the organization, and employees may be more inclined to choose this option. These findings form the basis for hypotheses H6a and H6b. Finally, in June 2022, the Malaysian Employers Federation announced in a media release that more than 90% of employees prefer a work-from-home arrangement, and effective September 2022, employees can apply for flexible working arrangements (FWA) [38]. As psychological, social, and situational factors yield a positive outcome towards work efficiency, work efficiency may have a positive impact on work-from-home preference; therefore, the following hypotheses are proposed:

Hypothesis 6b (H6a). Perceived organizational support is positively related to work efficiency.

Hypothesis 6b (H6b). Perceived organizational support is positively related to the preference for working from home.

Hypothesis 7 (H7). Work efficiency is positively related to the preference for working from home.

**Fig. 1:**
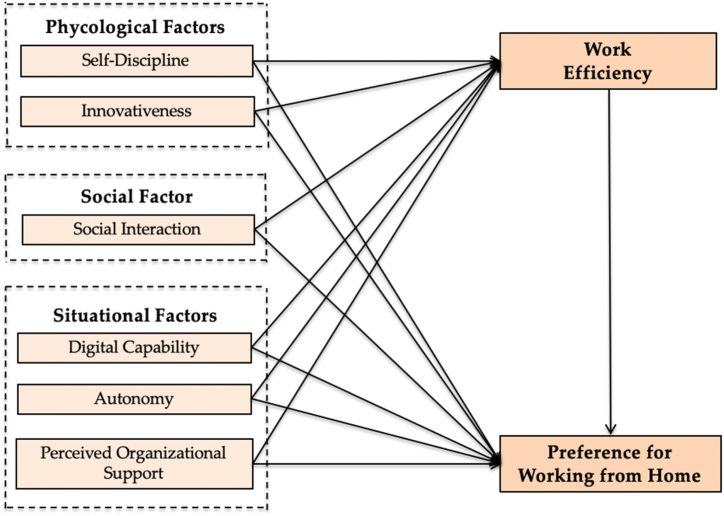
Research framework.

The research framework is presented in Fig. 1.

## 3 Methodology

### 3.1 Population and sample

The participants were chosen using a convenience sampling approach to enable access from any segment and region of the population that is easily available [39]. Hair et al. [40] suggests a sample size of at least 200 units when using partial least squares structural equation modelling (PLS-SEM). However, the minimum sample size was accurately determined using the G*power tool (version 3.1.9). (*f*^*2*^) refers to the effect size in statistical analysis, indicating the strength of a relationship or difference between groups. It provides a measure of how much variability in the dependent variable can be explained by the independent variable, helping researchers understand the practical significance of their findings [42]. Alpha probability (α) is a significance level used in hypothesis testing to determine the likelihood of observing a result by chance. It is typically set at 0.05, indicating a 5% chance of obtaining a result as extreme as the observed one due to random variation alone [43]. Setting the parameters as effect size (*f*^*2*^) = 0.15, α prob = 0.05, power (1-β err prob) = 0.95, and number of predictors = 7, the tool indicated that the sample size should include at least 153 respondents [41]. This study randomly collected responses from 288 working adults in China and Malaysia, because both these countries are developing Asian countries whose industrial structures are dominated by manufacturing industries and they also have similar GDP per capita [44].

Human Research Ethics Committee of Changzhi University approved this study (CZ-2022-0095). This study has been performed in accordance with the Declaration of Helsinki. Written informed consent for participation was obtained from respondents who participated in the survey.

### 3.2 Data collection

An online survey was conducted between September and December 2022 using a structured questionnaire that was distributed through online channels, such as WeChat, WhatsApp, and Facebook. This survey aimed to assess the impact of the COVID-19 pandemic on work from home, as suggested by local authorities amid the fluctuation in the death rate in Southeast Asia [45]. To qualify for the survey, the respondents had to meet the following conditions: (1) they should be 18 years of age or older, (2) they should be working full-time, and (3) their job should allow them to work from home. The questionnaire was published on WJX (https://www.wjx.cn) and Google Forms (https://www.google.com/forms). After eliminating the outliers, 288 responses were included in the statistical data analysis. The respondents’ personal information was kept confidential throughout the survey.

### 3.3 Measurement items

The questionnaire was developed by tailoring the questionnaires from earlier studies to fit the parameters of the current research goal. The questions were worded clearly, explicitly, and objectively to encourage the participants to share their honest opinions. The sample items used to measure self-discipline by Wang et al. [9] using the three-item scale adapted from Lindner, Nagy, and Retelsdorf [46] are “I do certain things that are bad for me if something is fun”, “I have trouble concentrating”, and “Sometimes I can’t stop myself from doing something, even if I know it is”. The items from Janssen [47] and Karani et al. [10] were used to assess innovativeness such as idea generation: sample item is “I am able to generate fresh solutions for challenging problems”. As well as idea realization: “I can introduce innovative ideas into the work environment in a systematic way”. Social interaction was evaluated by modifying the items from Winslow et al. [11]. A sample item is “I enjoyed interacting with people in my organization”. Digital capability was assessed using the items from Afrianty et al. [13]. A sample item is “I have the ability to work remotely away from the office”. Autonomy was measured by adapting the items from Patterson et al. [14] sample questions are like “I am allowed to make the decision without seeking permission first” and “My supervisor has tight control over me”. Items designed by Eisenberger et al. [12] were used to measure perceived organizational support. Such as: “My organization cares about my opinions”. And “My organization really cares about my well-being”. Work efficiency was determined using questions from Rožman and Čančer [48]. A sample question is “I do not feel a decrease in concentration when doing my work”. Finally, the preference for working from home was gauged by adapting the items from Gadot et al. [49]. The sample questions are “I prefer to work from home.” And “I can exercise my talents, even I am working from home”. Responses from the participants were collected using a Likert scale. All items presented as supplementary materials (*S1. Survey Questionnaire*). Complete data also submitted as supplementary materials (*S2. Dataset*). In this study, the Web Power tool was used for normality testing. The sample size was calculated using G*Power. Descriptive analysis and full collinearity test was performed using SPSS (V.29). SmartPLS (V.4) was used for structural equation modeling.

### 3.4 Multivariate normality

This study examined the multivariate skewness and kurtosis using the online statistical application, “Web Power” to evaluate the non-normality of all variables. The results of the study indicated that the p-values for both multivariate kurtosis and multivariate skewness were less than 0.05, which is the threshold suggested by Cain et al. [50]. This implies that the collected data were not normally distributed.

### 3.5 Data analysis methods

PLS-SEM was used as the data analysis technique for this study owing to the multivariate non-normality of the dataset. Sarstedt et al. [51] recommend the use of variance-based structural equation modelling to explore non-normal datasets and their dependent constructs. The SmartPLS (V4.0) software was used to evaluate the data. PLS-SEM is an exploratory method that considers integrated latent variable path correlations [52]. It is also a casual-predictive technique suitable for small datasets, and it does not require any specific assumptions of goodness-of-fit standards [51]. In addition, it enables the assessment of complex causal relationships between multiple components. Therefore, PLS-SEM was selected as the ideal data analysis technique for this study.

## 4 Findings

### 4.1 Demographic details

According to Table 1, the demographic characteristics of the respondents revealed that the majority (53.5%) of participants were female. Of the surveyed individuals, most were aged 26–30 years (20.5%), followed by those aged 18–25 years (20.1%), and 31–35 years (19.4%). With regards to marital status, 46.9% were married, 42.4% were single, and 9% were divorced. Regarding employment, 68.1% were employed in the private sector, 21.5% were employed in the public sector, and 10.4% were self-employed. Of the participants, 91.7% had no child(ren) under 4 years of age, while 8.3% lived with a child(ren) under 4 years of age. The highest educational attainment of the surveyed individuals was a bachelor’s degree or equivalent (41%), followed by those who had a diploma or technical school certificate (23.6%). Most participants (31.6%) earned between $2501 and $5000 per month; 20.5% earned between $5001 and $7500 per month, 17% earned between $7501 and $10,000, and 13.2% earned less than $2500 per month. Most participants (44.1%) had more than 10 years of work experience, followed by those who had been working for five years or less (36.8%). Of the respondents, 19.1% had 6–10 years of work experience. Among the respondents, 46.18% are from China and 53.82% are from Malaysia.

**Table 1:** Demographic characteristics.

	N	%		N	%
*Gender*			*Living with Infant*		
Female	154	53.5	Living with child(ren) below 4 years	24	8.3
Male	134	46.5	No child(ren) below 4 years	264	91.7
Total	288	100.0	Total	288	100.0
					
*Age Group*			*Education*		
18–25 Years	58	20.1	Secondary school certificate	38	13.2
26–30 Years	59	20.5	Diploma or technical school certificate	68	23.6
31–35 Years	56	19.4	Bachelor’s degree or equivalent	118	41.0
36–40 Years	53	18.4	Master’s degree	55	19.1
41–45 Years	29	10.1	Doctoral degree	9	3.1
46–50 Years	29	10.1	Total	288	100.0
51–55 Years	2	.7			
55–60 Years	1	.3	*Average Income*		
More than 60 Years	1	.3	<$2500	38	13.2
Total	288	100.0	$2501–$5000	91	31.6
			$5001–$7500	59	20.5
*Marital Status*			$7501–$10,000	49	17.0
Single	122	42.4	$10,001–$12,500	20	6.9
Married	135	46.9	>$12,501	31	10.8
Divorced	26	9.0	Total	288	100.0
Widowed	5	1.7			
Total	288	100.0	*Tenure*		
			5 Years or Less	106	36.8
*Sector*			6–10 Years	55	19.1
Public sector	62	21.5	More than 10 Years	127	44.1
Private sector	196	68.1	Total	288	100.0
Self-employed	30	10.4			
Total	288	100.0	*Country of Origin*		
			China	133	46.18
			Malaysia	155	53.82
			Total	288	100.0

**Note:** All currencies correspond to the respective countries of the respondents.

### 4.2 Common method bias

The single factor test proposed was applied to assess whether common method bias (CMB) affected the research model [53]. Podsakoff et al. [54] emphasized that if a single component accounted for over half of the variance, it would suggest an issue with CMB; however, this study found that the single component only accounted for 31.55% of the variance, and thus, CMB had no significant effect [55]. To ensure the absence of CMB, a full collinearity assessment was performed [55]. Table 2 shows that the variance inflation factor (VIF) values for all the constructions were below the cutoff of 3.3, as recommended by Kock [55]. This test confirmed the absence of problem concerning CMB in this data set.

**Table 2:** Full collinearity test.

Variables	Variance inflation factor
Self-Discipline	1.246
Innovativeness	1.995
Social Interaction	1.608
Digital Capability	2.292
Autonomy	2.154
Perceived Organizational Support	1.864
Work Efficiency	1.886
Preference for Working from Home	1.318

### 4.3 Measurement model (outer model)

Sarstedt et al. [51] emphasize that the measurement model should be assessed prior to the structural model. This assessment involves evaluating the internal consistency, reliability, convergent validity, and discriminant validity of the outer model to guarantee the robustness of the measurement model.

### 4.4 Internal consistency and convergent validity

Reliability determines the consistency of the scale’s measure structure [51], which can be determined through Cronbach’s alpha and composite reliability rho. As shown in Table 3, the values of both exceeded the threshold value of 0.7, indicating the reliability of the questionnaire’s internal consistency [40]. Furthermore, the average variance extracted (AVE) can be used to assess convergent validity, with Sarstedt et al. [51] recommending an AVE value of more than 0.5 for the model and its parts to ensure substantial convergent validity. The AVE values in Table 3 are in the range 0.596–0.748, which is above the threshold level and exhibits high convergent validity. The level of collinearity in the multiple linear regression models is accessed using the VIF index. The likelihood of commonality increased with a greater VIF rating among several factors. VIF typically has a value between 0.1 and 10, demonstrating a lack of conflict between the variables. The results of this study indicate that the VIF values ranged between 1.125 and 2.170, which do not exceed the suggested threshold of 5 proposed by Hair et al. [40]. Consequently, the VIF values were within the acceptable range.

**Table 3:** Reliability and validity.

Variables	No. of Items	Mean	Standard Deviation	Cronbach’s Alpha	Composite Reliability (rho_a)	Composite Reliability (rho_c)	Average Variance Extracted	Variance Inflation Factors
SD	3	3.072	0.885	0.727	0.889	0.829	0.621	1.125
IB	3	3.720	0.642	0.714	0.723	0.839	0.635	1.983
SI	3	3.742	0.708	0.814	0.818	0.889	0.728	1.620
DC	4	3.876	0.677	0.804	0.808	0.872	0.631	2.159
AM	3	3.716	0.667	0.722	0.728	0.843	0.642	2.170
PS	3	3.669	0.674	0.738	0.753	0.850	0.654	1.882
WE	4	3.582	0.685	0.776	0.781	0.855	0.596	1.943
WHP	4	4.638	1.251	0.889	0.919	0.922	0.748	–

**Note:** SD: Self-Discipline; IB: Innovativeness; SI: Social Interaction; DC: Digital Capability; AM: Autonomy; PS: Perceived Organizational Support; WE: Work Efficiency; WHP: Preference for Working from Home.

### 4.5 Discriminant validity

**Fig. 2:**
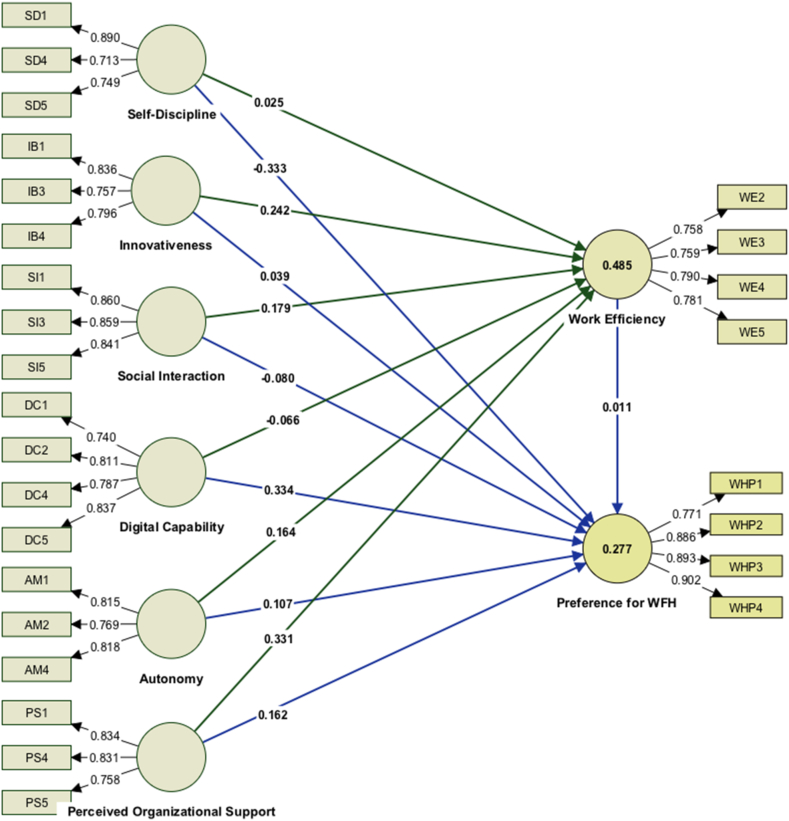
Research framework with findings.

To assess the discriminant validity of the model, the Fornell-Larcker criterion, Heterotrait-Monotrait (HTMT) ratio, and cross-loadings were evaluated. According to the Fornell-Larcker criterion, the square root of the AVE value for a construct should be larger than the variances of any other latent variables in the relevant row and column [51]. The results in Table 4 show that the Fornell-Larcker criterion values for each component are higher than any correlations in the corresponding row and column. Additionally, the HTMT values for all variables should be below 0.90 for high discriminant validity [56]. The HTMT values for each component in Table 4 are lower than 0.90, indicating that all the variables satisfy the maximum threshold. Lastly, cross-loading was used to identify the differences in the structures’ exterior loads and the values of all loadings; it should be higher than 0.60 to validate the high level of the model’s statistical validity [57]. In Appendix A, the factor loadings (also presented in Fig. 2) of each construct are shown in bold font, and they were found to be above the suggested minimum threshold. Consequently, the results of all three validity tests supported the strong discriminant validity of the model.

**Table 4:** Discriminant validity.

	SD	IB	SI	DC	AM	PS	WE	WHP
*Heterotrait-Monotrait Ratio (HTMT)*								
SD	–							
IB	0.174	–						
SI	0.211	0.662	–					
DC	0.317	0.789	0.588	–				
AM	0.409	0.779	0.618	0.862	–			
PS	0.326	0.694	0.611	0.671	0.706	–		
WE	0.238	0.741	0.635	0.568	0.698	0.767	–	
WHP	0.201	0.358	0.200	0.459	0.348	0.360	0.256	–
*Fornell-Larcker Criterion*								
SD	0.788							
IB	0.142	0.797						
SI	0.163	0.507	0.853					
DC	0.239	0.597	0.478	0.794				
AM	0.311	0.556	0.478	0.659	0.801			
PS	0.236	0.508	0.485	0.525	0.525	0.809		
WE	0.202	0.557	0.513	0.452	0.522	0.598	0.772	
WHP	−0.187	0.299	0.181	0.401	0.299	0.303	0.229	0.865

**Note:** SD: Self-Discipline; IB: Innovativeness; SI: Social Interaction; DC: Digital Capability; AM: Autonomy; PS: Perceived Organizational Support; WE: Work Efficiency; WHP: Preference for Working from Home.

### 4.6 Structural model (inner model)

Following the recommendations by Sarstedt et al. [51], this study assessed the structural model using path coefficient (β), coefficient of determination (*r*^*2*^), and effect size (*f*^*2*^). Bootstrapping was used to obtain p-values, t-values, and path coefficients for each relationship in the model.

### 4.7 Hypothesis testing

Table 5 presents the results of the hypotheses tests. The analysis indicates that the statistical value for self-discipline on work efficiency (β = 0.025, t = 0.519, p = 0.302) and for digital capability on work efficiency (β = −0.066, t = 0.952, p = 0.171), have no significant impact on work efficiency. Hence, the results indicate that hypotheses H1a and H4a are rejected. In addition to the statistical figures for innovativeness (β = 0.242, t = 4.041, p = 0.000) supporting the predictions, statistical values for social interaction (β = 0,179, t = 2.612, p = 0.005), autonomy (β = 0.164, t = 2.193, p = 0.014), and perceived organizational support (β = 0.331, t = 4.633, p = 0.000) also have a significant positive influence on work efficiency. Therefore, the results support hypotheses H2a, H3a, H5a, and H6a. On the other hand, statistical values for innovativeness (β = 0.039, t = 0.446, p = 0.328), social interaction (β = −0.080, t = 1.025, p = 0.153), and autonomy (β = 0.107, t = 1.296, p = 0.097), showed that the predictions on preference for working from home were not supported. Thus, hypotheses H2b, H3b, and H5b are rejected. Additionally, work efficiency (β = 0.011, t = 0.137, p = 0.445) was also reported to have no statistically significant preference for working from home, which also means that hypothesis H7 is rejected.

**Table 5:** Hypothesis testing.

Hypothesis		Beta	CI Min	CI Max	*t* Value	*p* Value	*f*^*2*^	*r*^*2*^	Decision
*Factors Effecting Work Efficiency*									
H1a	SD → WE	0.025	−0.052	0.107	0.519	0.302	0.001	0.277	Rejected
H2a	IB → WE	0.242	0.148	0.343	4.041	0.000	0.061		Supported
H3a	SI → WE	0.179	0.068	0.293	2.612	0.005	0.040		Supported
H4a	DC → WE	−0.066	−0.176	0.050	0.952	0.171	0.004		Rejected
H5a	AM → WE	0.164	0.042	0.287	2.193	0.014	0.025		Supported
H6a	PS → WE	0.331	0.214	0.448	4.633	0.000	0.127		Supported
*Factors Effecting Preference for Working from Home*									
H1b	SD → WHP	−0.333	−0.421	−0.248	6.286	0.000	0.136	0.485	Supported
H2b	IB → WHP	0.039	−0.108	0.181	0.446	0.328	0.001		Rejected
H3b	SI → WHP	−0.080	−0.204	0.054	1.025	0.153	0.005		Rejected
H4b	DC → WHP	0.334	0.194	0.473	3.943	0.000	0.072		Supported
H5b	AM → WHP	0.107	−0.030	0.243	1.296	0.097	0.007		Rejected
H6b	PS → WHP	0.162	0.002	0.308	1.742	0.041	0.019		Supported
H7	WE→ WHP	0.011	−0.122	0.149	0.137	0.445	0.000		Rejected

**Note:** SD: Self-Discipline; IB: Innovativeness; SI: Social Interaction; DC: Digital Capability; AM: Autonomy; PS: Perceived Organizational Support; WE: Work Efficiency; WHP: Preference for Working from Home.

Self-discipline had a significant influence on the preference for working from home with the following statistical values: β = −0.333, t = 6.286, and p = 0.000, thus supporting hypothesis H1b. Simultaneously, digital capability (β = 0.334, t = 3.943, p = 0.000) and perceived organizational support (β = 0.162, t = 0.137, p = 0.000) had a significant positive influence on the preference for working from home. Thus, the results confirm that hypotheses H4b and H6b are supported. Effect size, also known as *f*^*2*^, is a metric used to evaluate the extent to which exogenous factors have a significant impact on endogenous variables. This metric is derived from specific variance rather than from shared variances [51]. According to Cohen [58], the scale of the effects can be categorized as small (≥0.02), medium (≥0.15), and substantial (≥0.35). As the attributes of the framework and study area are distinct from one another, it may be challenging to verify the applicability of general guidelines to have a sizeable and noticeable influence [40]. The findings of the analysis regarding the influence size are small.

### 4.8 The coefficient of determination

The coefficient of determination (*r*^*2*^) is a metric that shows the extent to which the variance in the dependent variable is explained by a linear model. Endogenous latent variables are classified as strong, medium, or weak, depending on their *r*^*2*^ values, which are 0.75, 0.50, and 0.25, respectively [52]. Table 5 presents the *r*^*2*^ values for the two endogenous constructions in the study model. The *r*^*2*^ value of work efficiency (0.277) suggests that all related exogenous factors explained only 27.7% of the variation in work efficiency, indicating a weak explanatory power. Accordingly, the *r*^*2*^ values for the preference for working from home (0.485) indicate weak explanatory power through their related exogenous constructs.

### 4.9 Predictive assessment

The predictive capacity of the PLS model was evaluated using PLSpredict, where the effectiveness was measured based on the new forecasted observations. Most links showed strong predictive power as the Q^2^ Predict figures were in the range of 0 and 0.3 [59]. The predictive capability was examined by comparing the root mean squared error (RMSE) results from the PLS-SEM analysis and LM benchmark. Table 6 shows that the model has good predictive power [59].

**Table 6:** PLS predict.

Items	Q^2^ Predict	PLS-SEM RMSE	PLS-SEM MAE	LM RMSE	LM MAE	Difference (RMSE)	Difference (MAE)
WHP1	0.031	1.576	1.242	1.613	1.284	−0.037	−0.042
WHP2	0.246	1.217	0.984	1.253	0.986	−0.037	−0.002
WHP3	0.180	1.279	1.017	1.322	1.064	−0.043	−0.047
WHP4	0.178	1.253	0.986	1.280	1.005	−0.026	−0.019
WE2	0.226	0.802	0.642	0.818	0.646	−0.016	−0.004
WE3	0.218	0.819	0.638	0.841	0.660	−0.021	−0.021
WE4	0.283	0.723	0.569	0.737	0.581	−0.014	−0.012
WE5	0.330	0.703	0.542	0.709	0.542	−0.006	0.000

**Note:** WE: Work Efficiency; WHP: Preference for Working from Home.

## 5 Discussions

Interpreting the results suggests that factors that have a positive relation with work efficiency (innovativeness, social interaction, and autonomy) have a negative relation with a preference for working from home. Furthermore, factors that have a negative relationship with work efficiency (self-discipline and digital capability) have a positive relationship with preference for working from home. Thus, regarding H7, it is concluded that work efficiency is negatively related to the preference for working from home. However, perceived organizational support has a positive relationship with work efficiency and preference for working from home. This has the following implication for the managers: their ability to motivate employees is important for ensuring employees’ work efficiency as well as preference for working from home [60]. According to Brunelle [61], businesses and organizations must find new ways to manage remote employees, create fresh career paths, and implement support systems that are suitable for teleworkers. This study revealed the critical functions of virtual work features. Although more research is required, our results show that managers can improve remote workers’ productivity by providing perceived organizational support. In terms of digital capability, companies should create a digital atmosphere in which employers and staff can access the tools and applications required to run the business. This includes applications for tracking and monitoring health-related activities. Companies should promote the use of digital technologies and demonstrate how to configure them for maximum output in the least amount of time [62]. Furthermore, this study aligns with the findings of Pokojski et al. [60] in their study involving participants from Poland, which shows that work performance is not linked to a preference for working from home, as the authors suggest that employees view working from home as a temporary solution during the pandemic.

Human resource managers should provide training to the managers or recruit employees who can adapt to changes in their work culture. Maslow’s research on human needs has been brought to the forefront by the COVID-19 pandemic, highlighting the need for the management to provide social support to employees. Studies from the University of Maryland have revealed that employees feeling free to share their emotions with each other can lead to the development of inventive solutions to issues [63]. By endorsing an innovative work environment, businesses can motivate their employees to think ahead and exploit the available opportunities that result in groundbreaking innovations. The pandemic has triggered creativity in a multitude of areas in industry, and in the context of a completely remote workforce, these innovations have been strategized and implemented. Collaboration, job efficiency, and creativity can thrive when firms trust their employees’ autonomy while they work remotely, on-site, or in hybrid mode, and equip them with the correct combination of technologies [64]. In addition, the developing countries in Southeast Asia are relying heavily on manufacturing [6], which is labor intensive [7]; however, with the emergence of technologies, labor will soon be replaced. The introduction of artificial intelligence and 5G connectivity are known to be the drivers of the industrial revolution 4.0 [65]. In addition, based on an empirical study by Janssen [47], employees who work in Dutch food industries and have a higher education level, are more likely to control their jobs and are more involved in innovative activities. This finding is verified in this study as more than 60% of its participants have completed their tertiary education; this also indicates that innovative behavior has a direct impact on work efficiency.

Future studies could delve into other factors that may influence work efficiency and preferences for working from home. For example, factors such as work-life balance, organizational culture, leadership styles, task characteristics, and individual personality traits could be explored to gain a more comprehensive understanding of their impact on work efficiency in remote work settings. Examination of diverse outcomes of work efficiency: While this study focused on the preference for working from home as an outcome, future research could explore other outcomes related to work efficiency. For instance, studies could investigate the impact of work efficiency on employee well-being, job satisfaction, performance, creativity, and innovation in remote work environments. Understanding these relationships can provide valuable insights for managers and organizations in optimizing remote work arrangements.

## 6 Conclusions

The major findings of the study suggest that factors such as innovativeness, social interaction, and autonomy have a positive relationship with work efficiency but a negative relationship with the preference for working from home. On the other hand, factors like self-discipline and digital capability have a negative relationship with work efficiency but a positive relationship with the preference for working from home. The study concludes that work efficiency is negatively related to the preference for working from home. However, perceived organizational support has a positive relationship with both work efficiency and the preference for working from home. The implications of these findings for managers are significant. It highlights the importance of managers’ ability to motivate employees and provide perceived organizational support to ensure work efficiency and meet employees' preferences for remote work. Managers should focus on finding new ways to manage remote employees, create career paths suitable for teleworkers, and implement support systems tailored to remote work arrangements. The study emphasizes the critical role of virtual work features and suggests that managers can enhance remote workers' productivity by providing organizational support and creating a digital atmosphere that facilitates access to tools and applications necessary for efficient remote work. Additionally, the study aligns with previous research by highlighting that work performance is not necessarily linked to a preference for working from home. It suggests that employees may view remote work as a temporary solution during the pandemic. The findings emphasize the need for human resource managers to provide training to managers and recruit employees who can adapt to changes in work culture. The study also highlights the importance of social support, as employees’ freedom to share emotions can lead to innovative problem-solving. Trusting employees’ autonomy and equipping them with the right technologies can foster collaboration, job efficiency, and creativity in remote, on-site, or hybrid work settings.

This study responded to Jacks [15] call for the assessment of work from home productivity in the post-pandemic era. It has set a foundation for further research by exploring the situation in two developing countries in Southeast Asia, although following limitations must be noted. First, it would be interesting to investigate the preference for working from home in various industries. For instance, Malaysian Employers Federation reported more than 90% employees prefer work from home arrangement [38]; this study also found that more than 70% respondents from China and Malaysia either work in the private sector or are self-employed. It might be worth investigating whether employees from a particular industry have a higher preference for working from home or perceive themselves as being able to work remotely. Later, the top management from the relevant industry could actively seek support in terms of financial aid provided by the country, such as Industry 4.0 (Industry4WRD) Incentives, announced in Budget 2019 [66]. Through this program, the government aims to achieve growth in labor productivity, manufacturing contribution to the economy, innovative capacity, and high-skilled jobs. Therefore, it is suggested that future studies repeat this study while targeting a labor-intensive industry, such as the textile and garment industries [7].

This study relied on self-reported measures, which could lead to an overestimation of the results due to potential measurement errors [67]. Although common method bias has been addressed statistically, this limitation can be further examined by introducing a time delay in data collection [31]. To ensure the accuracy of the data, future research should use other sources of information such as colleagues and managers. Future studies should focus on senior and middle management to gauge their readiness for transition to remote work. In this study, perceived organizational support was the only factor influencing work efficiency and the preference for working from home, as the participants could perceive working from home as a temporary measure for the pandemic, and they might not be the decision makers empowered to determine or raise their concerns about wanting to work from home. In addition to China and Malaysia, a study by Pokojski et al. [60] targeted participants from Poland and faced the same questions: the participants perceived working from home as a temporary measure, as there is no clear direction if working from home can continue. Another limitation of this study is its narrow geographic scope, as it only included respondents from China and Malaysia. While these countries provided valuable insights into work-from-home practices in Southeast Asia, the findings may not fully represent the diversity of the region. Therefore, future studies should aim to include a broader representation of countries within Southeast Asia to obtain a more comprehensive estimate of the prevalence and characteristics of working people engaged in work-from-home arrangements. This would enhance the generalizability and applicability of the findings to the entire Southeast Asian population.

Finally, while self-discipline, social interaction, and digital capability are valuable factors in understanding human behaviour, it’s crucial to consider additional psychological, social, and situational factors. Psychological factors include motivation, personality traits, cognitive abilities, and emotional well-being. Social factors encompass cultural background, social support networks, and socioeconomic status. Situational factors involve environmental conditions, organizational structures, and access to resources. Exploring these factors further can provide a comprehensive understanding of behaviour, leading to improved interventions and strategies for areas like education, mental health, and workplace productivity.

## Ethical approval

Human Research Ethics Committee of Changzhi University approved this study (CZ-2022-0095).

## Availability of data and materials

The original contributions presented in the study are included in the article/Supplementary Material, further inquiries can be directed to the corresponding author/s.

## Funding

This research received no specific grant from any funding agency in the public, commercial, or not-for-profit sectors.

## Authors contribution

Wong May Yee, Zhong Xueyun, and Wan Mohd Hirwani Wan Hussain: Conceived and designed the experiments; Performed the experiments; Wrote the paper. Abdullah Al Mamun and Qing Yang: Performed the experiments; Analyzed and interpreted the data; Contributed reagents, materials, analysis tools or data; Wrote the paper. All authors approved the final version of the manuscript and give their consent for submission and publication.

## Declaration of competing interest

The authors declare that they have no known competing financial interests or personal relationships that could have appeared to influence the work reported in this paper.

## Figures and Tables

**Table:** 

	SD	IB	SI	DC	AM	PS	WE	WHP
SD1	0.890	0.163	0.140	0.199	0.308	0.187	0.236	−0.187
SD4	0.713	0.018	0.108	0.185	0.195	0.170	0.035	−0.129
SD5	0.749	0.097	0.139	0.193	0.193	0.216	0.125	−0.106
IB1	0.102	0.836	0.415	0.484	0.439	0.424	0.476	0.267
IB3	0.132	0.757	0.387	0.473	0.475	0.401	0.394	0.186
IB4	0.110	0.796	0.411	0.474	0.426	0.391	0.455	0.254
SI1	0.111	0.472	0.860	0.448	0.400	0.442	0.466	0.192
SI3	0.155	0.423	0.859	0.381	0.406	0.412	0.447	0.111
SI5	0.157	0.399	0.841	0.390	0.420	0.382	0.396	0.156
DC1	0.248	0.436	0.370	0.740	0.494	0.374	0.388	0.215
DC2	0.143	0.506	0.457	0.811	0.544	0.446	0.345	0.360
DC4	0.181	0.440	0.292	0.787	0.493	0.446	0.385	0.302
DC5	0.194	0.514	0.399	0.837	0.562	0.399	0.325	0.384
AM1	0.163	0.436	0.395	0.567	0.815	0.444	0.372	0.324
AM2	0.381	0.435	0.309	0.473	0.769	0.369	0.438	0.093
AM4	0.222	0.466	0.434	0.540	0.818	0.443	0.447	0.281
PS1	0.185	0.436	0.468	0.503	0.507	0.834	0.559	0.259
PS4	0.210	0.438	0.385	0.392	0.380	0.831	0.488	0.245
PS5	0.177	0.348	0.302	0.362	0.371	0.758	0.381	0.229
WE2	0.227	0.379	0.349	0.296	0.406	0.428	0.758	0.126
WE3	0.113	0.438	0.351	0.313	0.385	0.381	0.759	0.140
WE4	0.178	0.445	0.392	0.365	0.417	0.478	0.790	0.166
WE5	0.115	0.452	0.475	0.408	0.405	0.540	0.781	0.255
WHP1	−0.056	0.167	−0.009	0.246	0.145	0.158	0.057	0.771
WHP2	−0.254	0.314	0.198	0.372	0.280	0.279	0.198	0.886
WHP3	−0.133	0.258	0.186	0.368	0.294	0.309	0.251	0.893
WHP4	−0.155	0.261	0.185	0.371	0.277	0.269	0.238	0.902

**Note:** SD: Self-Discipline; IB: Innovativeness; SI: Social Interaction; DC: Digital Capability; AM: Autonomy; PS: Perceived Organizational Support; WE: Work Efficiency; WHP: Preference for Working from Home.
