# Supplementary material for: Modelling the significance of psychological, social, and situational factors on work efficiency and the preference for working from home in Southeast Asia
Source: Heliyon. 2023 Jun 23;9(6):e17561. doi: 10.1016/j.heliyon.2023.e17561 (PMC10289266; doi:10.1016/j.heliyon.2023.e17561)
Supplement: Multimedia component 1 [file mmc1.docx]

**Supporting Material - S1.** Survey Questionnaire

| Code | Questions |
| --- | --- |
| SD1 | I do certain things that are bad for me if something is fun. |
| SD4 | I have trouble concentrating. |
| SD5 | Sometimes I cannot stop myself from doing something, even if I know it is wrong. |
| IB1 | I am able to generate fresh solutions for challenging problems. |
| IB3 | I have the ability to get creative ideas approved. |
| IB4 | I am capable of turning original concepts into practical applications. |
| SI1 | I enjoyed interacting with people in my organization. |
| SI3 | My mood improved when I interacted with people in my organization. |
| SI5 | I felt good when people in my organization talked to me at work. |
| DC1 | I can find solutions to my problems on the Internet. |
| DC2 | I am proficient in using a variety of tools, including Microsoft Office, to work remotely. |
| DC4 | I am quick to pick up new software. |
| DC5 | I have basic technological understanding. |
| AM1 | I am allowed to make my own decision for most of the time. |
| AM2 | I am allowed to make the decision without seeking permission first. |
| AM4 | I have control over my actions. |
| PS1 | My organization cares about my opinions. |
| PS4 | Help is available from my organization when I have a problem. |
| PS5 | My organization would forgive an honest mistake on my part. |
| WE2 | I do not feel a lack of my work capacity. |
| WE3 | I do not feel a decrease in concentration when doing my work. |
| WE4 | I do not do less work than in normal working conditions. |
| WE5 | My willingness to work has not diminished. |
| WHP1 | I prefer to work from home. |
| WHP2 | I can exercise my talents, even I am working from home. |
| WHP3 | Most days I feel enthusiastic about my work when I work from home. |
| WHP4 | I feel fairly satisfied with my work when I work from home. |

**Note:** SD: Self-Discipline; IB: Innovativeness; SI: Social Interaction; DC: Digital Capability; AM: Autonomy; PS: Perceived Organizational Support; WE: Work Efficiency; WHP: Preference for Working from Home.
